# Regulation of the Human Phosphatase PTPN4 by the inter-domain linker connecting the PDZ and the phosphatase domains

**DOI:** 10.1038/s41598-017-08193-6

**Published:** 2017-08-11

**Authors:** Célia Caillet-Saguy, Angelo Toto, Raphael Guerois, Pierre Maisonneuve, Eva di Silvio, Kristi Sawyer, Stefano Gianni, Nicolas Wolff

**Affiliations:** 1 Institut Pasteur - CNRS, Unité de Résonance Magnétique Nucléaire des Biomolécules - UMR 3528, Département de Biologie Structurale et Chimie, Institut Pasteur, Paris, F-75724 France; 2 Istituto Pasteur - Fondazione Cenci Bolognetti and Istituto di Biologia e Patologia Molecolari del CNR, Dipartimento di Scienze Biochimiche “A. Rossi Fanelli”, Sapienza University of Rome, Rome, 00185 Italy; 3 Institute for Integrative Biology of the Cell (I2BC), CEA, CNRS, Université Paris-Sud, Université Paris-Saclay, Gif-sur-Yvette cedex, 91198 France

## Abstract

Human protein tyrosine phosphatase non-receptor type 4 (PTPN4) has been shown to prevent cell death. The active form of human PTPN4 consists of two globular domains, a PDZ (PSD-95/Dlg/ZO-1) domain and a phosphatase domain, tethered by a flexible linker. Targeting its PDZ domain abrogates this protection and triggers apoptosis. We previously demonstrated that the PDZ domain inhibits the phosphatase activity of PTPN4 and that the mere binding of a PDZ ligand is sufficient to release the catalytic inhibition. We demonstrate here that the linker connecting the PDZ domain and the phosphatase domain is involved in the regulation of the phosphatase activity in both PDZ-related inhibition and PDZ ligand-related activation events. We combined bioinformatics and kinetic studies to decipher the role of the linker in the PTPN4 activity. By comparing orthologous sequences, we identified a conserved patch of hydrophobic residues in the linker. We showed that mutations in this patch affect the regulation of the PTPN4 bidomain indicating that the PDZ-PDZ ligand regulation of PTPN4 is a linker-mediated mechanism. However, the mutations do not alter the binding of the PDZ ligand. This study strengthens the notion that inter-domain linker can be of functional importance in enzyme regulation of large multi-domain proteins.

## Introduction

Protein-tyrosine phosphatases (PTPs) represent the largest family of human phosphatases. These enzymes are critical in regulating signal transduction and their impairment has been previously associated with human diseases^1^. PTPN4 is a non-receptor tyrosine phosphatase whose multiple functions have been linked to T-cell signalling, learning, spatial memory and cerebellar synaptic plasticity^2–4^. Its overexpression reduces cell proliferation in COS-7 (CV-1 (simian) in Origin and carrying the SV40 genetic material) cells and suppresses CrkI-mediated cell growth and mobility in HEK293T (Human embryonic kidney) cells^5, 6^. Recently, PTPN4 has been identified as a specific inhibitor of the TIR-domain-containing adapter-inducing interferon-β-dependent toll-like receptor 4 pathway^7^.

**Figure 1:**
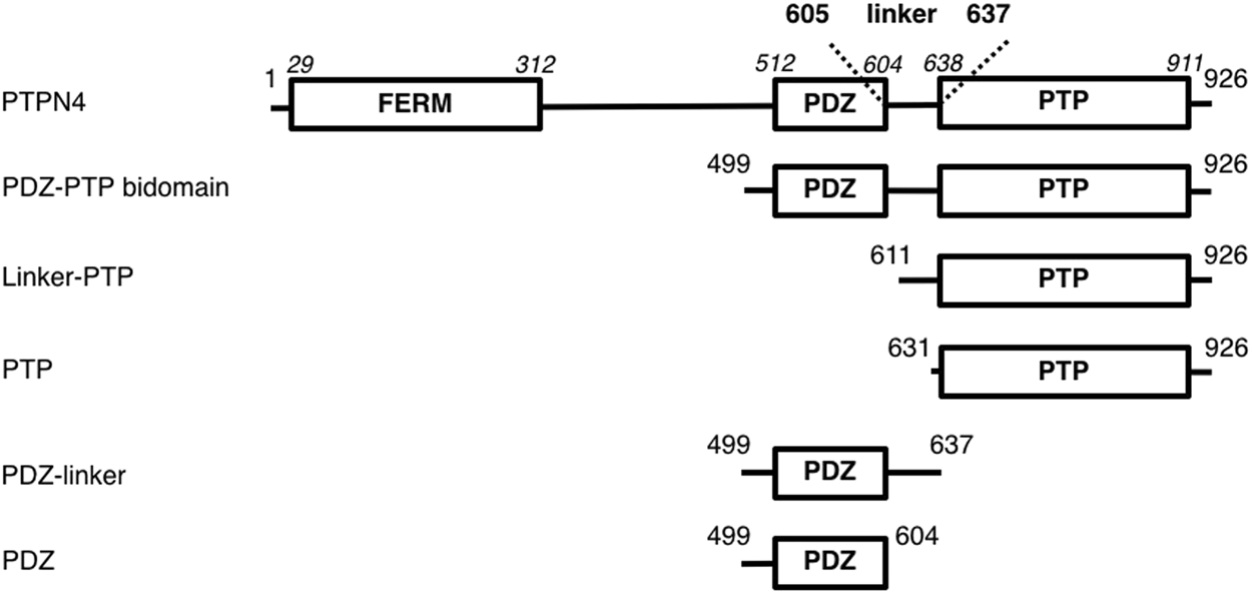
Schematic representation of the PTPN4 constructs. Numbers on both extremities of each schematic construct correspond to the boundary residues of the construct; Numbers in italic above the schematic construct of full-length PTPN4 correspond to the boundary residues of each protein domain.

From a structural perspective, PTPN4 (UniProt P29074) is a large modular protein containing a N-terminal FERM (Band 4.1, Ezrin, Radixin, and Moesin) domain, a PDZ (PSD-95/Dlg/ZO-1) domain and a C-terminal catalytic tyrosine phosphatase domain (Fig. 1). PTPN4 is localized in the cytoplasm and at the plasma membrane. After suppression of the FERM domain, the phosphatase is exclusively cytoplasmic^8^. PTPN4 is proteolysed in the cell by calpain in response to physiological stimuli, leading to enzyme activation^9^. *In vitro*, PTPN4 is cleaved and activated by both trypsin and calpain^9^. The active form of PTPN4 consists of the PDZ and PTP domains (Fig. 1).

We previously showed that PTPN4 prevents the induction of cell death in glioblastoma cell lines in a PDZ-PDZ ligand (or PDZ binding motif called PBM) dependent manner^10^. Targeting PTPN4-PDZ domain was shown to abrogate this protection and to trigger apoptosis. We recently identified the mitogen-activated protein kinase (MAPK) p38γ (also known as MAPK12) as a cellular partner of PTPN4. The PBM of p38γ has the highest affinity of all PBM from endogenous partners of PTPN4 and has a similar affinity to the optimized pro-death 13-amino acids peptide Cyto8-RETEV. Both peptides are efficient inducers of cell death after their intracellular delivery^11, 12^. Furthermore, we showed that the PDZ domain of PTPN4 inhibits the phosphatase activity, while a PBM bound to the PDZ domain abrogates the auto-inhibition of the catalytic activity of PTPN4^11, 13^.

While it is clear that the PDZ domain regulates the activity of PTPN4, the mechanism of crosstalk between the PDZ and PTP domains remains poorly understood and appears to be rather complex. In particular, previously published NMR (nuclear magnetic resonance) data indicated that the transition from the auto-inhibited state to the active state of PTPN4 is mediated by a modification of the global dynamic behaviour of the PDZ domain when going from the unbound to the bound state^13^. Of particular interest is the observation that, in order to communicate, the PDZ and PTP domains must be covalently linked as shown by the absence of inhibition when PTPN4-PDZ is added in trans to the linker-PTP construct^13^. These observations suggest that the linker has a crucial functional role in the communication between the two domains. Yet, the inter-domain linker of PTPN4 is flexible and unstructured, and does not display any direct detectable interactions by NMR with either the PDZ or the PTP domains^13^.

In this study, we investigate the role of the disordered linker connecting the PDZ and PTP domains in the active PDZ-PTP bidomain construct of PTPN4 (Fig. 1). To that end, the mechanism of regulation of PTPN4 activity was tackled both by bioinformatics and steady state kinetic experiments. We highlight a hydrophobic patch in the linker conserved among the PTPN4 orthologous sequences. Mutations in this conserved hydrophobic patch modify both the inhibition of the phosphatase activity of PTPN4 by the PDZ domain and the abrogation of the inhibition by the PBM binding. Our results shed light on the mechanism of PDZ-PBM mediated regulation of PTPN4 that is, at least in part, affected by the sequence of the linker.

## Results

### Structural and dynamics features of the linker

#### Bioinformatics analysis and structural features of the linker

**Figure 2:**
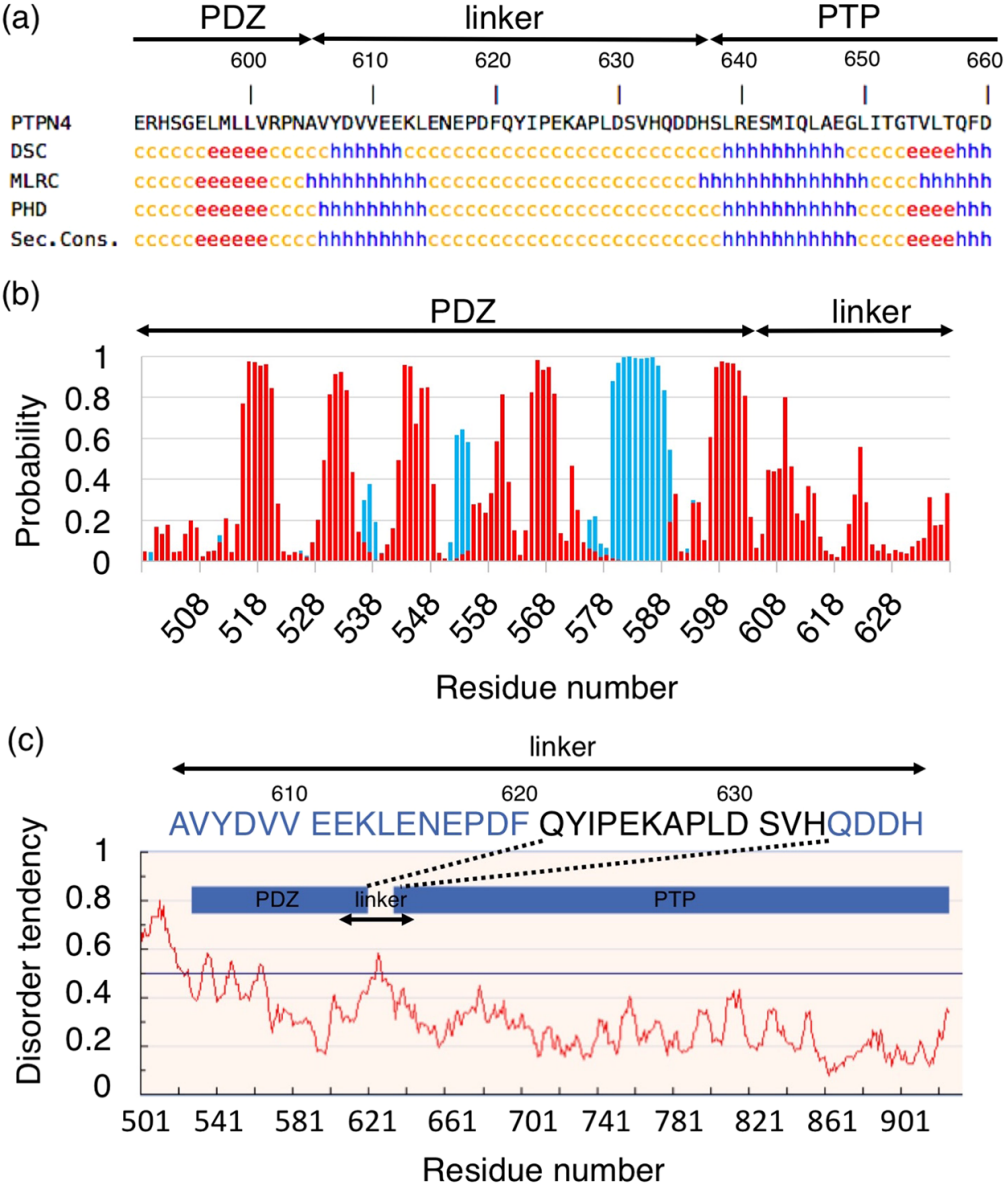
Secondary structure and unstructured region predictions of PTPN4 bidomain. (**a**) Linker sequence between the PDZ and PTP domains of PTPN4 (residues 605–637). Secondary structure predictions are shown below the sequence. For the different softwares used: h is the prediction of α-helix, e of β-strand and c to unstructured (http://npsa-pbil.ibcp.fr). (**b**) Secondary structure prediction of PDZ-linker construct of PTPN4 using Talos-N. Probability for the target residue to adopt secondary structure type α-helix or β-strand are shown in blue and red, respectively. (**c**) Unstructured region prediction of PDZ-PTP bidomain construct of PTPN4 using IUPred. Inter-domain linker (residues 605–635 of the linker) is indicated. Unstructured regions of the linker are shown in black on the sequence. Disorder tendency is shown below with the residue position.

PTPN4 PDZ-PTP active bidomain comprises two well-structured domains linked by an inter-domain sequence corresponding to residues 605 to 637 (Fig. 1). We previously stated that the linker was disordered by NMR but we also hypothesized that transient interactions operate in the bidomain. To access the structural features of the linker, the sequence of the linker was analysed using a panel of protein sequence analysis programs accessible from the NPS (network protein sequence) website for secondary structure predictions (https://npsa-prabi.ibcp.fr). Figure 2a shows the results obtained from different approaches – DSC^14^, MLRC^15^, PHD^16^ – and the consensus secondary structure prediction. Common features to all results are that a helix is predicted downstream of the PDZ domain (residues 605–614) and that the linker after residue 615 does not adopt any regular secondary structure. We have previously reported the resonance assignments of the linker (residue 605 to 637) for the PDZ-linker and the bidomain constructs (Fig. 1). Using the TALOS-N approach^17^ based on the resonance frequencies, the stretch of residues 605–614 is predicted as a β-strand with a low probability (<0.6) below the significant threshold (Fig. 2b). Thus, the linker is predominantly disordered in solution.

In addition, we used IUPred program from IUPred web server (http://iupred.enzim.hu)^18^ to determine the ordered or disordered characteristics of the linker in the PTPN4 PDZ-PTP. Interestingly, IUpred predicts a disorder tendency in the linker (residues 621 to 633) (Fig. 2c). Thus, in agreement with NMR results, bioinformatics predictors are consistent with an overall disordered conformation of the linker.

#### Protection of the linker from limited proteolysis and dynamic behaviour

**Figure 3:**
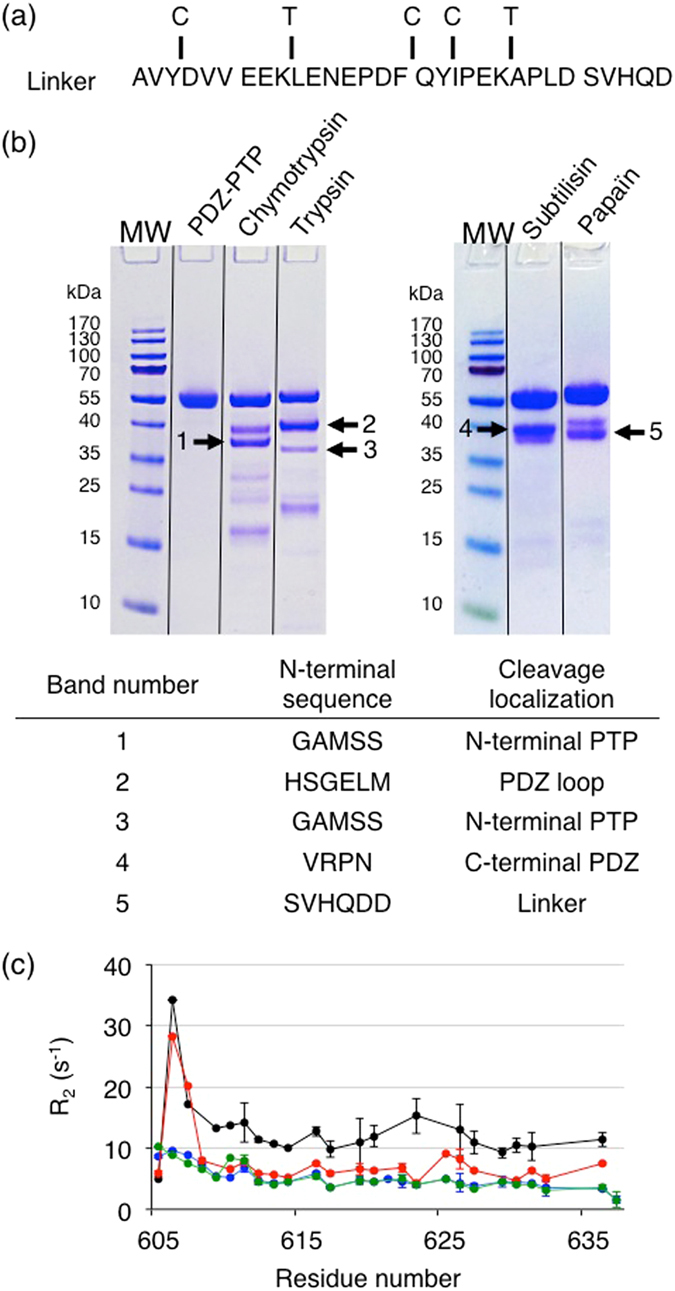
Limited proteolysis and dynamic behaviour of the linker. (**a**) Sequence of the linker of PTPN4 with the potential sites of cleavage of chymotrypsin and trypsin indicated by C and T, respectively. (**b**) Gel SDS-PAGE after one-hour incubation at 37 °C of PDZ-PTP_WT_ with proteases at a ratio (w:w) of 1:1000 for papain:PDZ-PTP_WT_ and subtilisin:PDZ-PTP_WT_, and 1:100 for trypsin:PDZ-PTP_WT_ and chymotrypsin:PDZ-PTP_WT_. Black lines indicate the grouping of the same gel. The most intense bands whose N-terminal sequences were identified by mass spectrometry are numbered. Identification of N-terminal sequences of the proteolytic fragments and cleavage localizations on PTPN4 are reported in the table. (**c**) Transverse ^15^N relaxation rates (R_2_) of the linker within several constructs of PTPN4: unliganded PDZ-linker (green), unliganded PDZ-PTP_C/S_ (black), PBM-liganded PDZ-linker (blue) and PBM-liganded PDZ-PTP_C/S_ (red). The PBM peptide used is Cyto8-RETEV.

To complement our results, we characterized PTPN4 PDZ-PTP by limited proteolysis with trypsin, chymotrypsin, and the non-specific subtilisin and papain proteases. Indeed, we identified three high specificity chymotrypsin cleavage sites and two trypsin cleavage sites in the linker from sequence analysis (Fig. 3a). The PDZ-PTP construct was subjected to limited proteolysis with different protein/protease ratio (100, 1000 and 10000) and different incubation times (0, 10, 60 min and overnight after addition of the protease) and at two different temperatures (18 °C and 37 °C). The limited proteolysis with a ratio 100 for chymotrypsin and trypsin and a ratio 1000 for subtilisin and papain after 60 min at 37 °C shown in Fig. 3b is representative of pattern also obtained with the other limited proteolysis conditions. The bands corresponding to the major proteolysis products were identified by mass spectrometry. Surprisingly, chymotrypsin, trypsin and subtilisin cleave only in the PDZ or PTP domains, while the linker is only cleaved by the non-specific papain in its C-terminal part. The observation that the linker is rather resistant to the proteolysis, suggests that the accessibility of this region is sufficiently low to protect it from the hydrolysis of peptide bonds. The linker-PTP construct is also rather resistant to the proteolysis (data not shown).

We previously collected dynamical parameters in solution of the bidomain PDZ-PTP_C/S_ (inactive enzyme by mutation of the catalytic cysteine) and of the PDZ-linker constructs both in the absence of PBM (unliganded) and with a PBM bound (liganded) to the PDZ domain. We reported that the transverse relaxation R_2_ and the longitudinal relaxation R_1_ values showed that the PDZ domain in the PDZ-PTP construct reorients more freely in solution when liganded with dynamic characteristics of the isolated PDZ domain^13^. We also collected dynamical parameters of the linker. R_2_ values are shown in Fig. 3c. The linker is flexible in the fast timescale (ps - ns) in both constructs. Interestingly, the linker (R_2_ of 6.5 s^−1^) is more flexible in the liganded bidomain PDZ-PTP_C/S_ than in the unliganded bidomain form (R_2_ of 13.0 s^−1^), with R_2_ values that tend toward the values observed in the PBM-liganded PDZ-linker isolated domain (R_2_ of 5.1 s^−1^). However, the linker flexibility in the liganded PDZ-PTP construct does not reach that of the isolated domain. Although we did not identify a stable surface of interaction between PDZ and PTP domains by NMR, from this dynamic behavior, we conclude that the transient intramolecular interactions that restrain the PDZ and the linker mobility in unliganded PDZ-PTP are destabilized in the PBM-liganded PDZ-PTP.

### Sequence alignment-based mutagenesis

**Figure 4:**
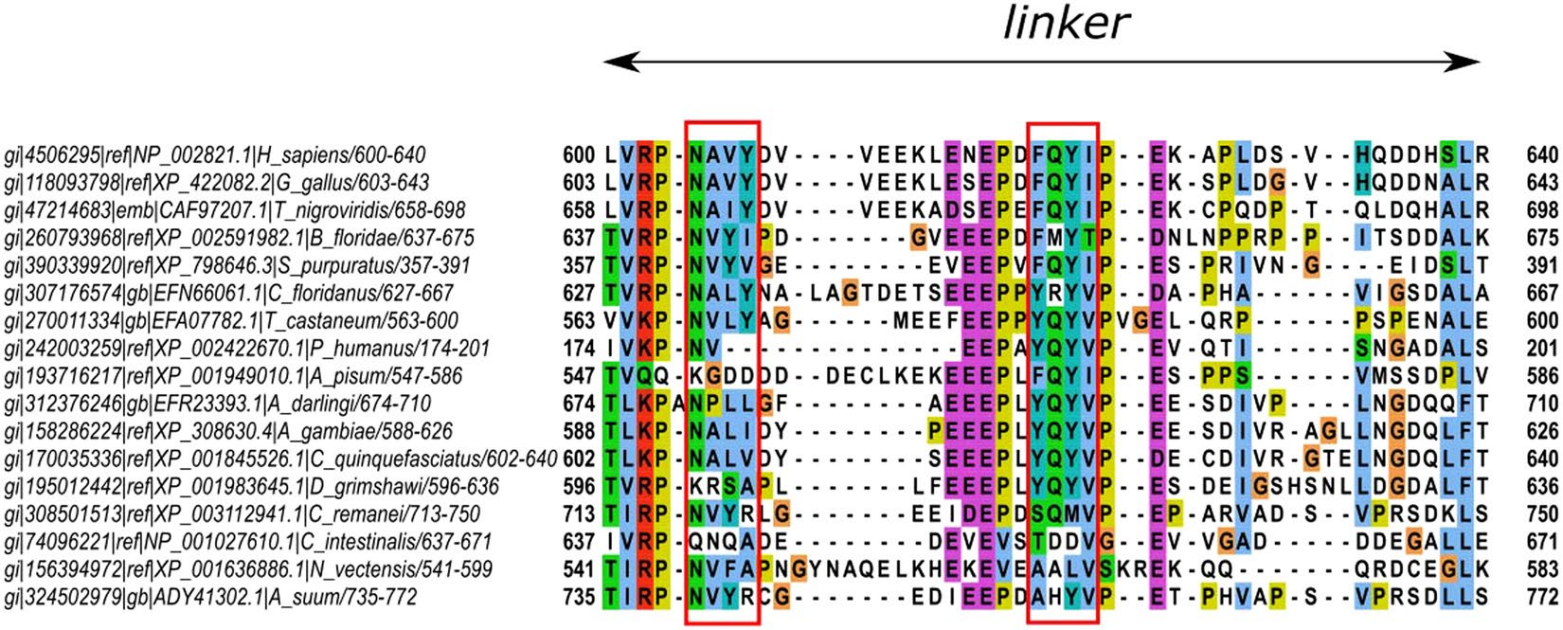
Sequence alignment of linker of PTPN4 from different species. The most conserved apolar motifs –NAVY– and – FQYI– are indicated in a red box.

To define a potential region of the linker involved in the PTPN4 regulation, the sequence of the linker of PTPN4 was aligned with orthologous sequences using the InterEvolAlign tool (http://biodev.cea.fr/interevol)^19^ that allows us to broaden the selection to homologous sequences and avoid paralogous sequences. We used a more divergent alignment with all proteins that have a PDZ-linker-PTP organization (Fig. 4).

We noticed two conserved stretches of hydrophobic residues in the linker. One, -NAVY-, is located at the N-terminus of the linker (residues 604–607) in the region predicted helical by secondary structure predictions programs (Fig. 4). We observed that in a sequence from lice (XP_002422670.1, *Pediculus humanus*) which presents the shortest linker (only 21 residues vs 35 residues in humans), this stretch becomes very short (two residues) (Fig. 4). Most interestingly, a more conserved and apolar motif – FQYI – is found in the central unstructured part of the linker (residues 620–623) (Fig. 4). We presumed that these highly conserved residues could be involved in the regulation of PTPN4 activity by interfacing with the PTP domain. We separately mutated the four positions by producing four site-directed glycine variants, namely F620G, Q621G, Y622G and I623G, which were analysed by stopped-flow kinetics. We also produced and analysed the mutant ΔFQYI with the deletion of the entire motif FQYI.

### Mutations in the inter-domain linker affect the phosphatase kinetics

In order to test the effect of sequence composition in the linker on the communication between the PDZ and PTP domains of PTPN4, we compared the steady-state kinetics of the bidomain wild-type PDZ-PTP construct (PDZ-PTP_WT_) to that of the F620G, Q621G, Y622G and I623G and ΔFQYI variants. First, we ensured that we obtained similar k_cat_ and K_M_ values for the linker-PTP that includes the inter-domain linker and the phosphatase domain, and the PTP construct that contains only the PTP domain (Fig. 1)(Table 1). This result indicates that the linker by itself had no effect on the PTP domain. Then, the linker-PTP construct was used both as the positive control for maximum activity and as the negative control for the regulation by the PDZ domain since no effect of PBM binding was observed on the k_cat_ and K_M_ values of the linker-PTP construct as previously reported for the k_cat_^13^ (Table 1).

**Table 1:** Kinetic parameters of the hydrolysis of pNPP by PTPN4 constructs.

PTPN4 constructs	*K*_M_ μM	*k*_cat_ s^−1^	*k*_cat_/*K*_M_ s^−1^.M^−1^
PTP	840 ± 60*	5.9 ± 0.2*	7000 ± 550*
linker-PTP	840 ± 130*	5.6 ± 0.5*	6700 ± 1200*
PDZ-PTP_WT_	230 ± 60	2.0 ± 0.3	8700 ± 1400
PDZ-PTP_F620G_	400 ± 100	1.3 ± 0.2	3300 ± 1000
PDZ-PTP_Q621G_	400 ± 100	1.8 ± 0.2	4500 ± 1500
PDZ-PTP_Y622G_	210 ± 40	1.0 ± 0.3	4800 ± 700
PDZ-PTP_I623G_	210 ± 40	1.5 ± 0.2	7100 ± 1000
PDZ-PTP_ΔFQYI_	70 ± 20	1.8 ± 0.4	25700 ± 10500
Linker-PTP + Cyto8-RETEV	780 ± 100*	6.0 ± 0.4*	7700 ± 1100*
PDZ-PTP_WT_ + Cyto8-RETEV	450 ± 70	2.9 ± 0.3	6500 ± 800
PDZ-PTP_F620G_ + Cyto8-RETEV	350 ± 90	2.0 ± 0.5	5700 ± 1800
PDZ-PTP_Q621G_ + Cyto8-RETEV	320 ± 60	2.5 ± 0.4	7800 ± 1400
PDZ-PTP_Y622G_ + Cyto8-RETEV	410 ± 80	1.5 ± 0.3	3500 ± 950
PDZ-PTP_I623G_ + Cyto8-RETEV	330 ± 70	1.9 ± 0.4	5800 ± 1400
PDZ-PTPΔFQYI + Cyto8-RETEV	290 ± 80	3.7 ± 0.9	12800 ± 4700

The experimental errors are derived from a bootstrap analysis except those with an asterisk that come from three independent experiments as described previously^13^.

The enzymatic activity was assessed by following the hydrolysis of the widely used phosphatase substrate p-nitrophenyl phosphate (pNPP).

#### Mutants of the linker without PBM peptides are functional with differential defects in catalytic inhibition

**Figure 5:**
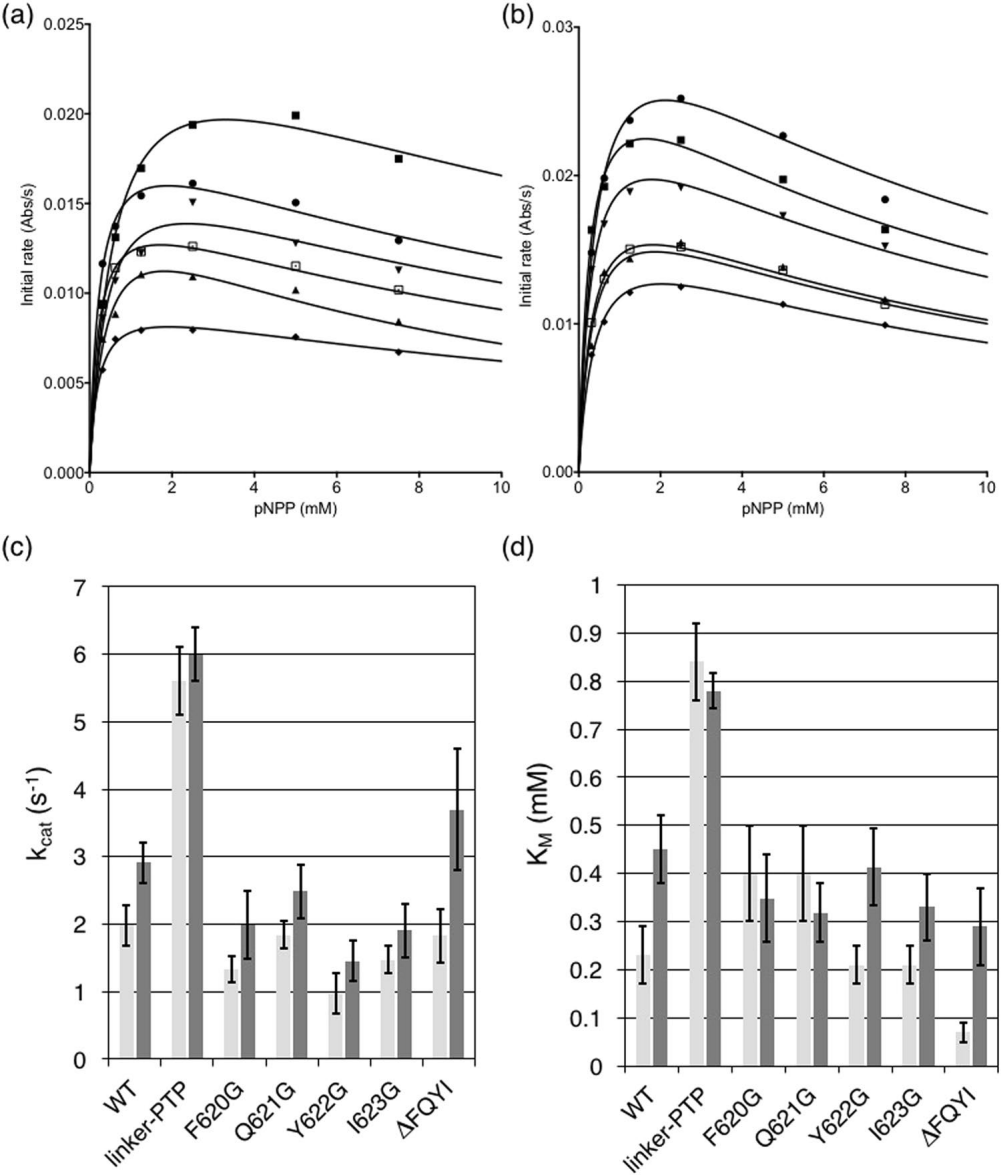
Phosphatase activity of PTPN4 bidomain constructs mutated in the linker. (**a**) Michaelis-Menten plots of initial rates of pNPP hydrolysis by different PTPN4 proteins at 600 nM without PDZ ligand; (**b**) Michaelis-Menten plots of initial rates of pNPP hydrolysis by different PTPN4 proteins at 600 nM in the presence of a saturated concentration of PDZ ligand (70–100 μM of Cyto8-RETEV);  linker-PTP;  PDZ-PTP_WT_;  F620G;  Q621G;  Y622G;  I623G. The solid lines are nonlinear least-squares fits of the data to the Michaelis-Menten equation. (**c**) k_cat_ of PTPN4 constructs in the absence of PDZ ligand (light grey) and in the presence of PDZ ligand (Cyto8-RETEV) (dark grey). (**d**) K_M_ of PTPN4 constructs in the absence of PDZ ligand (light grey) and in the presence of PDZ ligand (Cyto8-RETEV) (dark grey).

Kinetics experiments of PTPN4 bidomain constructs were first performed in the absence of PBM corresponding to the inhibited state of the phosphatase to determine catalytic parameters of the PTPN4 mutants. We showed that PDZ-PTP mutants dephosphorylate the pNPP substrate following Michaelis-Menten kinetics as previously reported for the PDZ-PTP_WT_ and linker-PTP (Fig. 5a). In all cases, we observed a clear substrate inhibition effect and, therefore, we employed a corrected Michaelis-Menten equation taking into account this effect. The Michaelis constant (K_M_) and the turnover number (k_cat_) values obtained by fitting the experimental data are listed in Table 1. The k_cat_ and K_M_ values decrease when comparing linker-PTP with PDZ-PTP_WT_ as previously reported^13^. K_M_ values of Y622G and I623G mutants are similar to the one of the PDZ-PTP_WT_ while those of F620G and Q621G are significantly higher by 1.7 fold, with intermediate values between those of PDZ-PTP_WT_ and linker-PTP (Table 1). All k_cat_ values are lower than PDZ-PTP_WT_ except for Q621G, which has a value similar to the wild-type. Y622G is strongly affected with a k_cat_ value divided by a factor 2 whereas F620G and I623G k_cat_ values decreased by 1.5 and 1.3 fold, respectively. Hence, it appears that, despite the fact that all the mutations are not involved directly in the function of the phosphatase domain, we could observe in all cases a significant effect in either the affinity of PTP substrate and/or the catalytic activity of the enzyme. Interestingly, the mutant ΔFQYI is strongly affected in the PTP-subtrate affinity with a K_M_ value of 70 μM, divided by a factor 3.3 compared to the PDZ-PTP_WT_ whereas the k_cat_ value is similar to the PDZ-PTP_WT_. The catalysis efficiency (k_cat_/K_M_ ratio) of ΔFQYI is thus strongly increased.

#### Mutations of the linker disturb PBM-mediated abrogation of catalytic inhibition

Kinetic experiments of PTPN4 PDZ-PTP constructs with increasing concentrations of PTPN4-optimized PBM Cyto8-RETEV^12^ peptide were performed to determine catalytic parameters of the bidomain mutants in their “activated” state (Fig. 5b).

Both k_cat_ and K_M_ values of PDZ-PTP_WT_ significantly increase, by a factor of 1.5 and 2, respectively, upon addition of PBM Cyto8-RETEV whereas the k_cat_ and K_M_ of the linker-PTP are unaffected^13^ (Table 1)(Fig. 5c). All k_cat_ values of PBM-liganded mutants are higher than those of non-liganded mutants (1.3 to 1.5 fold higher depending on single mutants) indicating that PBM abrogates inhibition for all mutants, but those values are lower than that of PBM-liganded PDZ-PTP_WT_ except for the PBM-liganded ΔFQYI mutant that presents a k_cat_ 2 fold higher. Thus, all mutants are affected in the PBM-mediated release of inhibition (Fig. 5c). However, all PDZ-PTP mutants bind Cyto8-RETEV with the same affinity as previously reported for PDZ-PTP_WT_ (Kd between 1 and 5 μM deduced from the fit of Vm as a function of Cyto8-RETEV concentration). Interestingly, K_M_ of F620G and Q621G are unchanged upon the addition of Cyto8-RETEV whereas K_M_ of Y622G and I623G increase (1.9 and 1.5 fold, respectively) as observed with the K_M_ of the wild-type (Fig. 5d). As previously observed for the PDZ-PTP_WT_, both the k_cat_ and K_M_ values significantly increase in the presence of Cyto8-RETEV for Y622G and I623G, showing that the release of the inhibition operates with a partial compensation of the effect on the two rate constants (Table 1) providing catalysis efficiencies (k_cat_/K_M_ ratios) that are not strongly changed in the presence or absence of the effector peptide.

Hence, all PDZ-PTP mutants are affected in the PBM-mediated regulation of PTPN4 activity either in the modulation of the affinity for the PTP substrate or in the catalytic activity of the enzyme. This modulation of the PDZ-mediated inhibition and PBM-mediated release of inhibition of the PTPN4 bidomain suggests that the linker has a function in the inter-domain PDZ-PTP communication driving the regulation of the activity of the phosphatase PTPN4.

## Discussion

Previous structural investigation of PTPN4 revealed that this enzyme is regulated by a mechanism of auto-inhibition which is, at least in part, mediated by an intramolecular communication between the PDZ and PTP domains^13^. Remarkably, the linker region connecting the PDZ and PTP domains is required for such a crosstalk, as the regulation is abolished when the two domains are not covalently linked. However, the 33-residue linker is flexible and unstructured, and no detectable interaction could be observed by NMR with either the PDZ or the PTP domains. These findings prompted us to perform additional investigations, in order to characterize more directly the role of the linker in the regulation of PTPN4.

It is interesting to observe that, whilst the linker is largely disordered^13^, it is not fully solvent accessible, displaying resistance to proteolysis in both PDZ-PTP and linker-PTP constructs and that NMR dynamics parameters (R_1_ and R_2_) do not reach the values of the truncated PDZ-linker construct. This suggest a potential interaction between the linker and the PTP domain. We previously proposed that the transient intramolecular interactions which constrain the PDZ and the linker mobility in unliganded PTPN4 are destabilized in the presence of the PBM. Based on multiple sequence alignment, we identified a conserved hydrophobic region in the linker that may be involved in the regulation of PTPN4, being universally conserved in PTPN4 from different species.

We showed that mutations in this hydrophobic patch –FQYI–, highly conserved among the PTPN4 orthologous sequences, affects the phosphatase kinetics with effects on k_cat_ and/or K_M_. Both the inhibition of the phosphatase activity by the PDZ domain and the abrogation of the inhibition by the PBM binding are affected, indicating that the PDZ-PBM regulation of PTPN4 is a linker-mediated mechanism.

**Figure 6:**
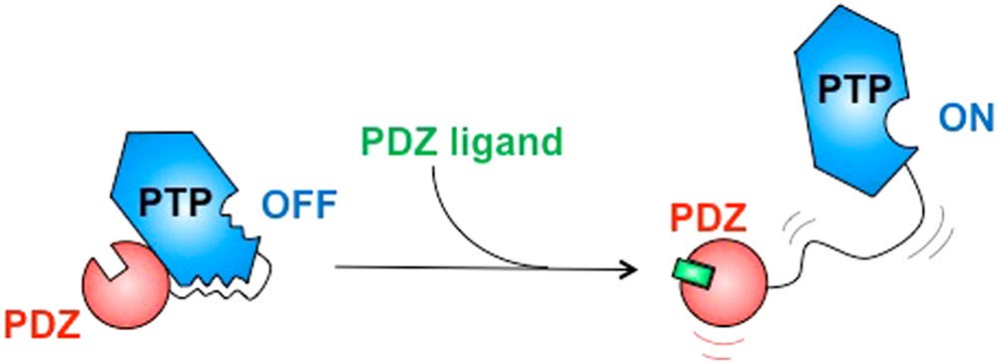
Schematic representation of the catalytic regulation of PTPN4. Transient interactions between linker, PDZ and PTP domains allosterically inhibit the catalytic activity of PTPN4. The binding of PDZ ligand partially disrupts these interactions and consequently activates the phosphatase.

We reported that the PDZ domain inhibits the adjacent catalytic PTP domain through a mixed inhibition mechanism, affecting the K_M_ and k_cat_ of the enzyme with opposite effects. The PBM binding to the PDZ domain abolishes this auto-inhibition and restores the catalytic properties of PTPN4, most probably affecting the active site via a distal interaction occurring through a protein interface. So, the PDZ domain of PTPN4 is not only directly involved in discriminating its PBM-containing targets but also allosterically regulates the enzymatic activity of the phosphatase through the PDZ-PTP linker. Thus, both the linker sequence and PDZ domain are required for the regulation of the enzymatic activity of PTPN4 (Fig. 6). Interestingly, such dynamic inter-domain regulation has been observed for the peptidyl-prolyl cis-trans isomerase Pin1. Substrate binding to Pin1 WW domain changes the intra/inter domain mobility, thereby altering substrate activity in the distal peptidyl-prolyl isomérase catalytic site with evidence of dynamic allostery^20^.

We propose that the linker participate together with the PDZ domain to the allosteric control of the catalytic activity by modulating the kinetic properties of the conserved WPD loop motif closure. Indeed, substrate binding is accompanied by a rotation of the WPD loop from an open to a closed position around the phosphotyrosine residue of the substrate that provides residues that are essential for the catalytic cycle^21^. Moreover, an allosteric regulation of the WPD loop closure was recently detailed in PTP1B showing a modulation of the activity with a likely enhancement of the phosphate release step in the PTP1B catalytic cycle^22^. This type of modulation of the WPD loop is consistent with the kinetic parameters of PTPN4. Indeed, a lower K_M_ for the unliganded PDZ-PTP_WT_ is related to the open state offering a larger accessibility of the substrate to the catalytic site, while the higher K_M_ and k_cat_ for the liganded PDZ-PTP_WT_ is related to the PBM binding that would promote both an enhancement of the activity and a decrease in substrate accessibility by triggering the closure of the WPD loop. The F620G and Q621G mutants completely lose the regulation on the K_M_ upon PBM binding in agreement with this proposal. Altogether, our data strongly suggest a pathway through the linker for the mechanism of PDZ-based regulation of PDZ-PTP bidomain with transient inter-domain interactions partially destabilized upon PBM binding.

## Methods

### Site-directed mutagenesis and protein purification

The variants of PTPN4 named F620G, Q621G, Y52G, I623G and ΔFQYI were obtained by using the Quik-Change Mutagenesis kit (Stratagene) according to the manufacturer’s instructions, and the substitutions were confirmed by DNA sequencing. Template DNA used for PTPN4 mutants was the PDZ-PTP_WT_ construct cloned in a pET15b expression plasmid. The PTP construct was obtained by using the Pwo polymerase (Roche) according to the manufacturer’s instructions, and the insertion was confirmed by DNA sequencing. Template DNA used for the PTP construct was a synthetic construct comprising the PTP domain cloned in a pgex6p1 expression plasmid. Expression and purification of linker-PTP and PTP construct were performed as described previously^13^. Bidomain PDZ-PTP variants were expressed and purified as previously described without TEV cleavage. Briefly, clarified supernatants were loaded on Ni^2+^ column (HiTrap Chelating HP, GE), washed and eluted with an imidazole gradient from 40 mM to 1 M. The eluted fractions containing the protein were pooled and loaded on a gel filtration column (Hiload Superdex 75 pg, GE). Proteins were concentrated using centrifugal filter devices (Vivaspin, Sartorius). Protein concentration was estimated from its absorbance at 280 nm. The peptide Cyto8-RETEV (SAESHKSGRETEV) was synthesized in solid phase using a Fmoc strategy (JPT).

### Limited proteolysis

Limited proteolysis was carried out in 50 mM Tris pH 7.5, 150 mM NaCl and 0.5 mM TCEP (Tris(2-carboxyethyl)phosphine hydrochloride) using a ratio (w:w) of 1:1000 for papain:PDZ-PTP_WT_ and subtilisin:PDZ-PTP_WT_, and 1:100 for trypsin:PDZ-PTP_WT_ and chymotrypsin:PDZ-PTP_WT_ at 37 °C for 0, 10, 60 min and overnight. Reaction mixtures at each time interval were stopped by mixing with SDS loading buffer and heating at 95 °C for 5 min. Protein fragments were resolved by SDS-PAGE through a 12% polyacrylamide gel and visualized by Coomassie staining. The bands corresponding of the major proteolysis products were then identified by matrix-assisted laser desorption/ionization mass spectrometry (MALDI-MS).

### Sequence alignment

Sequence of PTPN4 (accession number NP_002821.1) was used as query on the InterEvolAlign server^19^ to retrieve one single homolog per species assessed as probable ortholog through a reciprocal blast search procedure against the non-redundant database. Retrieved full-length sequences were re-aligned using MAFFT^23^ and displayed using Jalview^24^.

### Enzymatic assays

Kinetics experiments were carried out on a single-mixing SX-18 stopped-flow instrument (Applied Photophysics). Absorbances values were measured continuously at 410 nm at 25 °C. Experiments were performed by mixing a constant concentration of each of the PTPN4 variants, 0.6 μM in the presence of increasing concentrations of Cyto8-RETEV peptide, ranging between 0 μM and 100 μM versus increasing concentrations of pNPP ranging from 19 μM to 10 mM. The buffer was 50 mM Tris-HCl, pH 7.5, 1 mM MgCl2, 150 mM NaCl, 0.5 mM TCEP. Initial linear reaction rates were calculated during a 20 s reaction. The *K*_M_ and *k*_cat_ constants were deduced from fitting the Michaelis–Menten equation with the prism software. The equation takes into account excess substrate inhibition.
